# Isolated or concomitant tricuspid valve surgery for massive or torrential tricuspid regurgitation

**DOI:** 10.1016/j.xjon.2026.101944

**Published:** 2026-06-27

**Authors:** Hironobu Sakurai, Satoshi Kainuma, Naonori Kawamoto, Kizuku Yamashita, Kota Suzuki, Takashi Kakuta, Ayumi Ikuta, Rieko Kutsuzawa, Yuki Tadokoro, Kazuki Miyatani, Shinichi Kurashima, Yuki Irie, Kenji Moriuchi, Masashi Amano, Atsushi Okada, Makoto Amaki, Hideaki Kanzaki, Takeshi Kitai, Chisato Izumi, Kazuhiro Yamamoto, Satsuki Fukushima

**Affiliations:** a Department of Cardiac Surgery, National Cerebral and Cardiovascular Center, Suita, Osaka, Japan; b Department of Cardiovascular Medicine, National Cerebral and Cardiovascular Center, Suita, Osaka, Japan

**Keywords:** tricuspid valve, tricuspid regurgitation, tricuspid valve repair, tricuspid valve replacement

## Abstract

**Objective:**

Massive or torrential tricuspid regurgitation is associated with poor survival; however, its impact on surgical results remains uncertain. Outcomes following tricuspid valve surgery in patients with severe versus massive or torrential tricuspid regurgitation were compared.

**Methods:**

From 2010-2024, 164 symptomatic patients (mean age 71.2 ± 10.4 years) with severe (n = 69, 42%) or massive/torrential tricuspid regurgitation (n = 95, 58%) underwent tricuspid valve surgery, with or without concomitant procedures. Postoperative tricuspid regurgitation grade was the primary endpoint. Mortality and heart failure hospitalization predictors were analyzed. The mean follow-up was 4.6 ± 3.9 years.

**Results:**

Tricuspid repair was performed more frequent in patients with severe than with massive/torrential tricuspid regurgitation (96% vs 80%, *P* = .004). In-hospital mortality rates were 0% and 2.1%, respectively (*P* = .506). Postoperatively, tricuspid regurgitation improved significantly in both groups, with similar rates of residual tricuspid regurgitation mild or less (73.9% vs 72.3%, *P* = .930). During the follow-up period, 26 patients died and 38 were re-hospitalized for heart failure. Five-year survival was similar between the groups (85% vs 81%, *P* = .358). Higher left ventricular ejection fraction (adjusted hazard ratio 0.97, *P* = .027) and estimated glomerular filtration rate (adjusted hazard ratio 0.98, *P* = .016) were independently protective for adverse outcomes, whereas baseline tricuspid regurgitation severity was not.

**Conclusions:**

Tricuspid valve surgery for severe or massive/torrential tricuspid regurgitation can be performed with low perioperative mortality, and provides favorable long-term outcomes. Prognosis is determined primarily by baseline cardiac and renal function, rather than preoperative tricuspid regurgitation severity.

## Graphical Abstract

**Figure:**
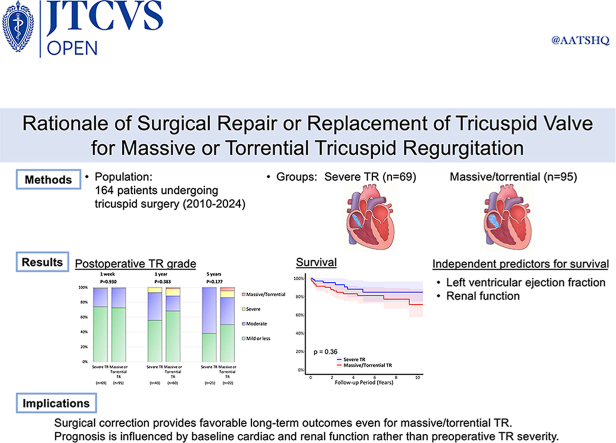

**Figure:**
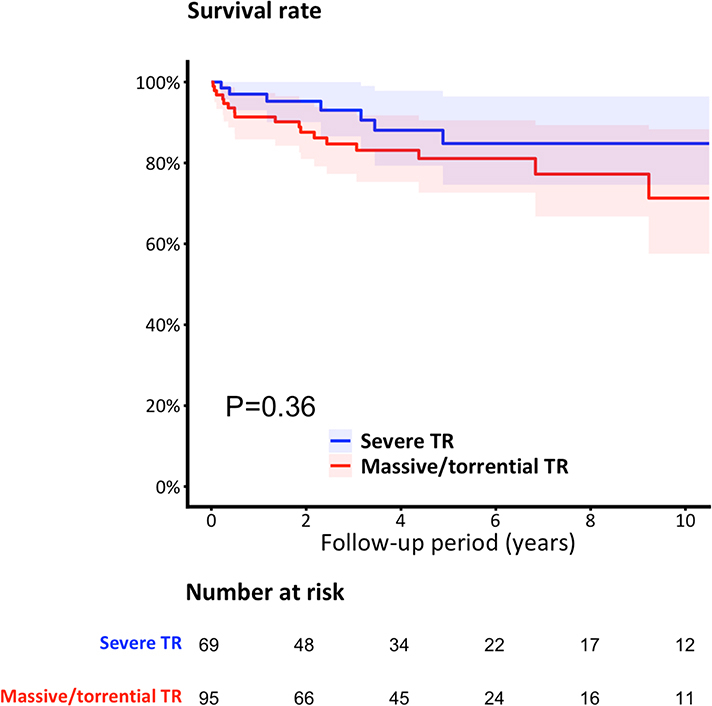
Survival rate after tricuspid surgery in patients with severe or massive/torrential TR.

**Central Message**

Patients with severe or greater TR can be safely and effectively treated with surgery. Prognosis is often influenced by baseline cardiac contractile function and renal function.

**Perspective**

For patients with severe or massive/torrential tricuspid regurgitation (TR), tricuspid valve surgery can be safely performed and leads to significantly improved TR grade. Preoperative impaired left ventricular and renal functions, but not preoperative TR severity, were found to be associated with a higher risk of mortality and/or heart failure following surgery.

Tricuspid regurgitation (TR) is a progressive disease associated with right-sided volume overload, systemic venous congestion, and multiorgan dysfunction, leading to significant morbidity and mortality.^1^, ^2^, ^3^ Once symptoms develop, prognosis on the basis of medical therapy alone is poor, with high rates of heart failure hospitalization and death reported.^4^^,^^5^ Therefore, current guidelines from major societies recommend timely intervention for significant TR.^6^, ^7^, ^8^ Concomitant tricuspid valve surgery is recommended or reasonable in patients with severe TR undergoing left-sided valve surgery (Class I/IIa). For isolated TR, surgery is recommended or reasonable in patients with severe primary TR who are symptomatic or in those with severe secondary TR and recurrent heart failure despite optimal medical therapy, provided advanced right ventricular (RV) dysfunction or irreversible pulmonary hypertension is absent (Class I or IIa). Transcatheter intervention may be considered at experienced centers for symptomatic secondary severe TR in patients deemed inoperable (Class IIb). Recently, severe TR has been divided into severe, massive, and torrential, with earlier treatment emphasized to prevent reaching an advanced stage, because progressively worse outcomes associated with increasing TR severity have been consistently shown in natural history studies.^9^

Advances in perioperative management and surgical techniques have led to improved surgical outcomes as well as increased interest in the operative correction of TR.^10^, ^11^, ^12^ However, most related studies have focused on conventionally defined severe TR, and the impact of massive or torrential TR severity on surgical outcomes remains unclear. Whether similar risk gradients exist after surgical correction and preoperative TR grade itself is an independent factor related to postoperative outcome are issues that remain to be elucidated.

Although RV and hepatic dysfunction have been identified as adverse prognostic markers after tricuspid surgery, the prognostic significance of left ventricular (LV) function, renal impairment, and surgical strategy (repair vs replacement) in contemporary cohorts remains to be elucidated.^13^ Clarification of these determinants is critical for optimizing patient selection and intervention timing, especially with the ongoing evolution of less-invasive transcatheter approaches. The present study was performed to evaluate clinical and echocardiographic outcomes after tricuspid valve surgery in patients with severe TR as compared with those with massive/torrential TR.

## Methods

**Figure 1:**
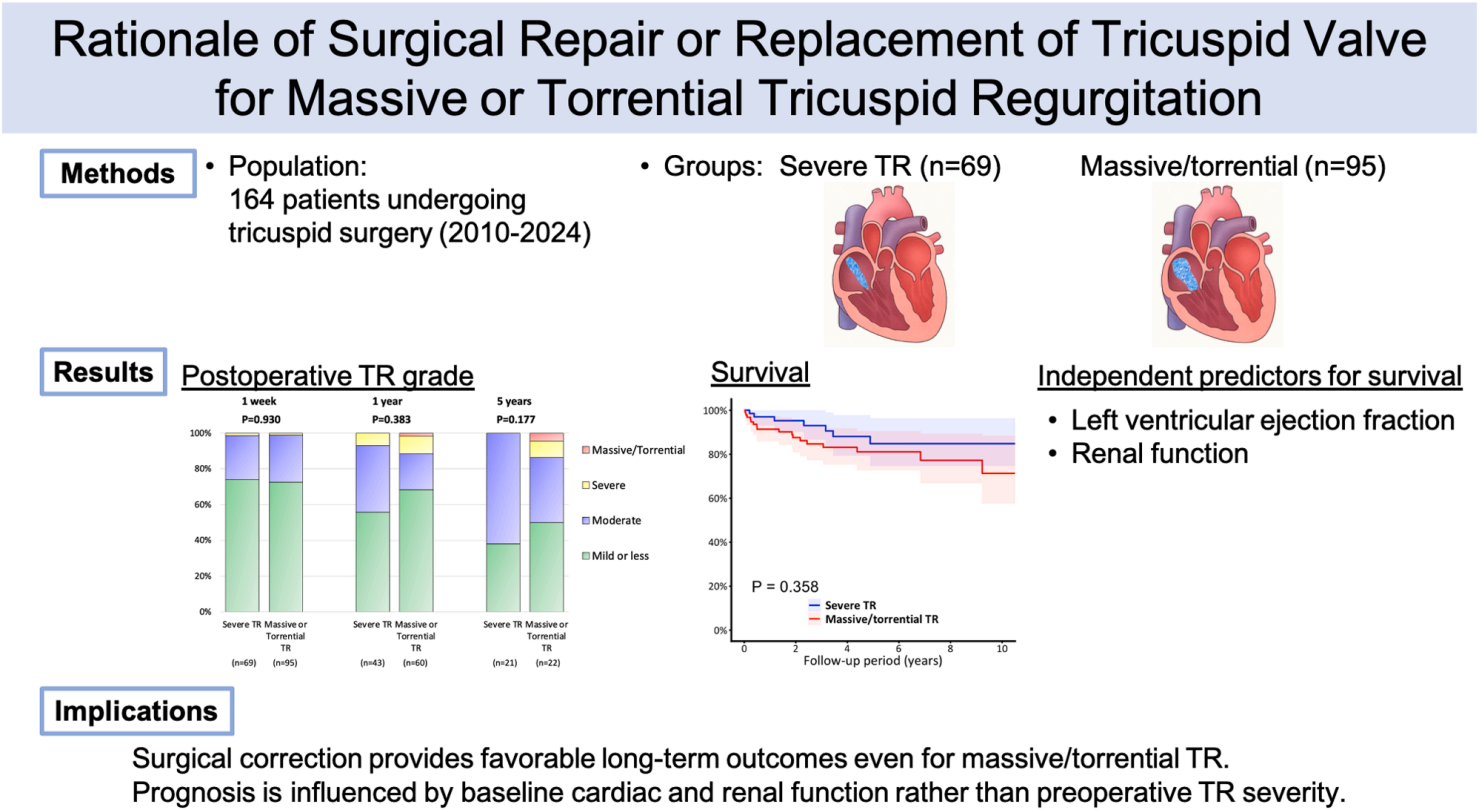
Tricuspid valve surgery improves regurgitation with favorable long-term outcomes, even in massive/torrential tricuspid regurgitation; prognosis is determined by cardiac and renal function. *TR*, Tricuspid regurgitation.

**Figure E1:**
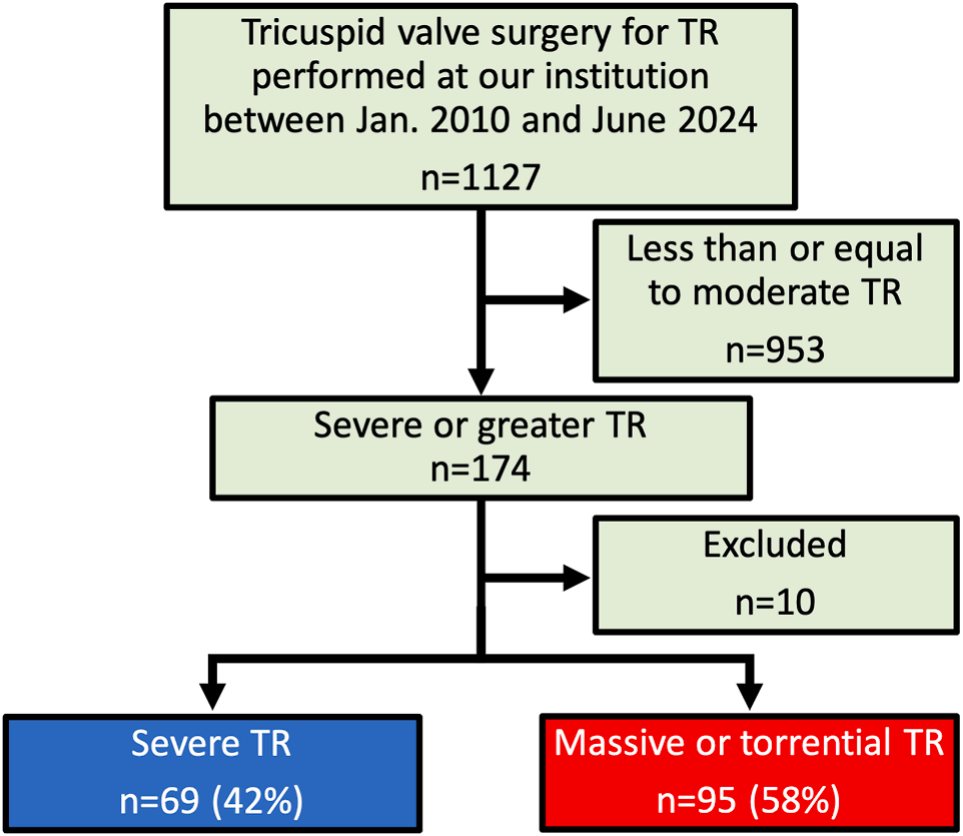
Patient selection flow chart. *TR*, Tricuspid regurgitation.

The study design, patient groups, and key outcomes are summarized in Figure 1. The institutional surgical database of the National Cerebral and Cardiovascular Center hospital was examined, which included a consecutive series of 1127 patients with TR who underwent tricuspid valve surgery between January 2010 and August 2024. Of those, 953 with TR graded as moderate or less were excluded, whereas an additional 10 patients were excluded because they underwent concomitant intracardiac repair for congenital heart disease or implantation of a ventricular assist device was planned. Thus, for the present study 164 patients (mean age 71 ± 11 years, 46% male) with severe (n = 69, 42%) or massive/torrential TR (n = 95, 58%) who underwent tricuspid valve repair or replacement were enrolled (Figure E1).

### Ethics Statement

The procedures for data collection, analysis, and reporting were approved by the institutional review board of the National Cerebral and Cardiovascular Center (reference no. M30-026; approval date: March 25, 2022). Each of the enrolled patients provided written informed consent for release of their related information.

### Surgical Techniques

All surgical procedures were performed with the patient under general anesthesia using a cardiopulmonary bypass procedure, via either a sternotomy (n = 125, 76%) or right thoracotomy approach (n = 39, 24%). Tricuspid valve repair was attempted whenever possible, regardless of the TR etiology, with ring annuloplasty as the primary technique to address annular dilatation. Additional repair procedures were performed according to valve pathology, including pacemaker lead relocation for lead-related TR and leaflet plication for anatomical leaflet abnormalities. More recently, repair techniques such as papillary muscle approximation or leaflet patch augmentation were used when annuloplasty alone was insufficient. Replacement was favored when the valve showed excessive degeneration and shortening. The final decision was made at the discretion of the attending surgeon on the basis of clinical condition and TR grade.

### Echocardiography

Two-dimensional and Doppler echocardiography examinations were performed by experienced cardiologists before (baseline) surgery, at 1 week and 6 months after surgery, and then annually thereafter, with anatomic and Doppler measurements obtained according to the American Society of Echocardiography recommendations. TR severity was determined on the basis of vena contracta width, with severe TR defined as 7 to 14 mm and massive/torrential TR as ≥14 mm.^5^ This approach follows previously validated grading schemes in which vena contracta width is used to distinguish severe TR from more advanced disease stages.^10^^,^^12^

Additional RV parameters, including tricuspid annular diameter and right RV fractional area change, were available in most patients evaluated after 2019; however, these data were incomplete in earlier cases because of the limited availability of stored echocardiographic clips. Therefore, these parameters were not included in the primary analysis and are presented as supplementary data.

### Group Classification

The enrolled patients were divided into 2 groups on the basis of baseline TR severity; 69 (42%) in the severe TR group (vena contracta width 7-14 mm) and 95 (58%) in the massive/torrential TR group (vena contracta width ≥14 mm).

### Clinical End Points and Follow-up

All-cause mortality was the primary end point, whereas the secondary end points were postoperative TR grading and a composite of mortality and/or heart failure readmission. The composite of all-cause mortality and/or heart failure readmission would capture clinically meaningful events related to residual or recurrent valvular dysfunction and ventricular function after surgery.

Clinical follow-up examinations were completed after a mean duration of 4.6 ± 3.9 years, with a maximum of 14.8 years, totaling 758 patient-years. Follow-up was complete for all patients, with survival status or last clinical contact available in all 164 cases. The decreasing number at risk at later time points reflects the inclusion of patients with shorter postoperative follow-up as well as the occurrence of death or heart failure hospitalization events during follow-up. Transthoracic echocardiography at 5 years was available in 43 patients.

### Statistical Analysis

Continuous variables are presented as mean ± SD or median (interquartile range) values, as appropriate. Results of comparisons between the 2 groups were evaluated using the Mann-Whitney *U* test or Wilcoxon rank sum test. Categorical variables are presented as counts and proportions and were compared using the Fisher exact test or a χ^2^ test, as appropriate. Survival curves and freedom from composite events were determined using the Kaplan-Meier method, and the results compared with an overall log-rank test. Echocardiographic variables over time were analyzed using a linear mixed-effects model for repeated measures, with group, time, and group-by-time interaction included as fixed effects. Patient was included as a random effect and assessment time points were used as categorical factors. An unstructured variance-covariance matrix in the linear mixed-effects model was assumed.

The Cox proportional hazard model was used to assess independent determinants for all-cause mortality. Results are summarized as adjusted hazard ratios (HRs) with 95% CIs. Probability values are presented as 2-sided. All statistical analyses were performed using R, version 4.5.2 (R Core Team, 2025).

## Results

### Patient Demographics

Baseline demographic and preoperative echocardiographic characteristics of the enrolled patients are summarized in Table 1. Surgical indications were TR in 52 patients, mitral regurgitation in 57, mitral stenosis in 17, mitral stenosis with regurgitation in 15, mitral prosthetic valve dysfunction in 7, infective endocarditis in 5, and other indications in 11. The prevalence of massive or torrential TR was similar between patients with moderate or greater mitral regurgitation (46/77, 60%) and those with less-than-moderate mitral regurgitation (49/87, 56%), with no significant difference between groups (*P* = .752). Among the 52 patients with TR as the primary surgical indication, 28 underwent isolated tricuspid valve surgery, whereas the remaining 24 underwent concomitant procedures, including mitral valve surgery in 21 patients, aortic valve replacement in 2 patients, and coronary artery bypass grafting in 1 patient. Anatomical abnormality was defined as valvular morphologic variants, such as leaflet cleft or anomalous chordal attachment, without associated congenital cardiac structural anomalies. Fifty-four patients had previously undergone cardiac surgery. Two patients had previously undergone tricuspid annuloplasty at the time of left-sided valve surgery. Reoperation was performed as the result of progressive TR in 1 patient and progression of concomitant mitral regurgitation in the other.

**Table 1:** Patient demographics

Severity	Tricuspid surgery					
	Isolated tricuspid surgery (n = 34)			Concomitant tricuspid surgery (n = 130)		
	Severe TR (n = 13)	Massive/torrential TR (n = 21)	*P* value	Severe TR (n = 56)	Massive/torrential TR (n = 74)	*P* value
Clinical variables						
Age, y	66.6 ± 16.1	70.3 ± 10.9	.433	72.8 ± 6.7	71.0 ± 11.2	.303
Male sex, n (%)	10 (77%)	8 (38%)	.039	21 (38%)	36 (49%)	.217
BSA, m^2^	1.64 ± 0.24	1.55 ± 0.17	.253	1.50 ± 0.17	1.54 ± 0.17	.227
NYHA functional class, n			.729			.002
I	2 (15%)	4 (19%)		2 (4%)	0 (0.0%)	
II	8 (62%)	9 (43%)		46 (82%)	47 (64%)	
III	2 (15%)	8 (38%)		7 (13%)	20 (27%)	
IV	1 (7.7%)	0 (0.0%)		1 (1.8%)	7 (9.5%)	
Inotrope or MCS, n	1 (7.7%)	0 (0.0%)	.382	1 (1.8%)	7 (9.5%)	.137
Hepatic dysfunction, n∗	0 (0.0%)	1 (4.8%)	1.000	6 (11%)	8 (11%)	1.000
eGFR, mL/min/1.73 m^2^	55 (42-66)	41 (31-61)	.218	48 (37-56)	49 (39-64)	.459
Brain natriuretic peptide, pg/mL	118 (44-608)	226 (82-456)	.753	114 (80-225)	237 (89-411)	.026
EuroSCORE	3.7 (1.3-7.9)	3.4 (1.7-4.3)	.701	2.9 (2.2-7.4)	3.1 (1.9-7.0)	.778
Comorbidities, n						
Diabetes mellitus	3 (25%)	0 (0.0%)	.048	13 (23%)	8 (11%)	.091
Hypertension	6 (46%)	6 (29%)	.462	28 (50%)	24 (32%)	.049
Dyslipidemia	6 (46%)	5 (24%)	.262	17 (30%)	11 (15%)	.051
Atrial fibrillation	5 (38%)	14 (67%)	.160	49 (88%)	66 (89%)	.788
History of pacemaker implantation	5 (38%)	4 (19%)	.254	3 (5.4%)	3 (4.1%)	1.000
Previous cardiac surgery, n	6 (50%)	7 (35%)	.491	21 (37%)	20 (27%)	.253
Echocardiographic findings						
Etiology of TR						
Primary TR, n	6 (46%)	13 (62%)	.484	6 (11%)	11 (15%)	.603
Infective endocarditis	1 (7.7%)	0 (0.0%)		1 (1.8%)	1 (1.4%)	
Device-related	2 (15.4%)	4 (19%)		1 (1.8%)	3 (4.1%)	
Prolapse	1 (7.7%)	5 (24%)		0 (0.0%)	3 (4.1%)	
Anatomical abnormality	2 (15.4%)	3 (14%)		3 (5.4%)	4 (5.4%)	
Posttricuspid surgery	0 (0.0%)	1 (4.8%)		1 (1.8%)	0 (0.0%)	
Secondary TR	7 (54%)	8 (38%)		50 (89%)	63 (85%)	
LV function parameters						
LV ejection fraction, %	56 ± 11	59 ± 9	.398	58 ± 9	54 ± 11	.019
LV end-diastolic dimension, mm	44 ± 9.5	42 ± 8.9	.518	49 ± 7.3	52 ± 8.5	.035
LV end-systolic dimension, mm	30 ± 7.6	28 ± 8.8	.547	33 ± 6.1	35 ± 8.0	.032
LA dimension, mm	43 ± 14.5	47 ± 11.8	.466	59 ± 12.4	59 ± 11.3	.954
TR severity						
Vena contracta, mm	11 ± 2.0	22 ± 8.0	<.01	11 ± 1.8	21 ± 5.6	<.01
MR moderate or greater, n	0 (0.0%)	1 (4.8%)	1.000	31 (55%)	45 (61%)	.592

*TR*, Tricuspid regurgitation; *BSA*, body surface area; *NYHA*, New York Heart Association; *MCS*, mechanical circulatory support; *eGFR*, estimated glomerular filtration rate; *EuroSCORE*, European System for Cardiac Operative Risk Evaluation; *LV*, left ventricle; *LA*, left atrium; *MR*, mitral regurgitation.

∗ Hepatic dysfunction: total bilirubin >2.0 mg/dL.

There were no significant differences between the severe TR and massive/torrential groups for age, sex, prevalence of atrial fibrillation, hepatic dysfunction (defined as preoperative total bilirubin level ≥2 mg/dL), estimated glomerular filtration rate (eGFR), or previous cardiac surgery. In contrast, the prevalence of diabetes mellitus, hypertension, and dyslipidemia was significantly greater among patients with severe TR. Although the mean European System for Cardiac Operative Risk Evaluation II was comparable between the groups (severe 3.05, massive/torrential 3.19; *P* = .739), patients with massive/torrential TR exhibited greater plasma brain natriuretic peptide levels (79 vs 89 pg/mL, *P* = .035) and greater incidence of heart failure symptoms, as reflected by New York Heart Association class III or IV (16% and 37%, respectively; *P* < .01).

Primary TR was observed in 17% and 24% of patients in the severe and massive/torrential groups, respectively (*P* = .256), whereas LV dimensions, LV ejection fraction, and left atrial dimensions were comparable between them. Vena contracta width was significantly greater in patients with massive/torrential TR (11.3 ± 1.9 vs 20.7 ± 6.2 mm, *P* < .01). However, the prevalence of concomitant moderate or greater mitral regurgitation was not significantly different between the groups (45% vs 48%, *P* = .709).

### Intraoperative Characteristics

Intraoperative characteristics are summarized in Table 2. Tricuspid valve replacement was performed in 3 patients (4%) with severe TR and in 19 (20%) with massive/torrential TR, whereas valve repair was performed in 66 (96%) and 76 (80%), respectively (*P* = .004). Among the 22 patients who underwent valve replacement, biological prostheses were used in all except for one in the severe group. The primary repair technique used was ring annuloplasty, with additional procedures, such as artificial chordae implantation, leaflet patch augmentation, or leaflet plication, performed in 7 patients in the severe group and 15 in the massive/torrential group.

**Table 2:** Surgical data

Severity	Tricuspid surgery					
	Isolated tricuspid surgery (n = 34)			Concomitant tricuspid surgery (n = 130)		
	Severe TR (n = 13)	Massive/torrential TR (n = 21)	*P* value	Severe TR (n = 56)	Massive/torrential TR (n = 74)	*P* value
Tricuspid valve surgery n (%)						
Replacement	3 (23%)	10 (48%)	.276	0 (0.0%)	9 (12%)	.010
Tissue valve	2 (15.4%)	10 (48%)		0 (0.0%)	9 (12%)	
Mechanical valve	1 (7.7%)	0 (0.0%)		0 (0.0%)	0 (0.0%)	
Repair, n	10 (77%)	11 (52%)		56 (100%)	65 (88%)	
Annuloplasty	8 (62%)	11 (52%)		56 (100%)	63 (85%)	
Other techniques, n (%)	4 (31%)	7 (33%)		3 (5.4%)	8 (11%)	
Patch augmentation	1 (7.7%)	1 (4.8%)		1 (1.8%)	2 (2.7%)	
Leaflet plication	2 (15.4%)	3 (14%)		0 (0.0%)	4 (5.4%)	
Artificial chordae	0 (0.0%)	4 (19%)		2 (3.6%)	1 (1.4%)	
Vegetation removal	1 (7.7%)	0 (0.0%)		0 (0.0%)	1 (1.4%)	
Others	1 (7.7%)	0 (0.0%)		0 (0.0%)	2 (2.7%)	
Operation time, min	269 ± 91	316 ± 135	.271	310 ± 103	321 ± 114	.601
Cardiopulmonary bypass time, min	139 ± 73	152 ± 82	.635	160 ± 52	169 ± 73	.431
Crossclamp time, min	76 ± 18	90 ± 57	.512	114 ± 37	113 ± 48	.931
Right minithoracotomy, n	6 (46%)	7 (33%)	.491	13 (23%)	13 (18%)	1.000
Concomitant surgery, n (%)						
Aortic valve	–	–		14 (20%)	12 (13%)	.270
Mitral valve	–	–		51 (74%)	69 (73%)	.745
Maze procedure	3 (23%)	8 (38%)	.465	13 (23%)	10 (14%)	.170
LAA closure	2 (15%)	8 (38%)	.251	28 (50%)	39 (53%)	.860
CABG	1 (7.7%)	1 (4.8%)	1.000	3 (5.4%)	2 (2.7%)	.651
PFO/ASD closure	2 (15%)	2 (9.5%)	.627	3 (5.4%)	7 (9.5%)	.514
Pacemaker implantation	1 (7.7%)	8 (38%)	.107	3 (5.4%)	9 (12%)	.231
Others	2 (15%)	6 (29%)	.444	12 (21%)	15 (20%)	1.000
Postoperative outcomes						
Death within 30 d	0 (0.0%)	0 (0.0%)	1.000	0 (0.0%)	2 (2.7%)	.506
Complication within 30 d						
Readmission for heart failure	1 (7.7%)	0 (0.0%)		1 (1.8%)	1 (1.4%)	
Stroke	0 (0.0%)	0 (0.0%)		2 (3.6%)	0 (0.0%)	
Myocardial infarction	0 (0.0%)	0 (0.0%)		1 (1.8%)	2 (2.7%)	
Low output syndrome	0 (0.0%)	0 (0.0%)		0 (0.0%)	2 (2.7%)	
Re-exploration for bleeding	0 (0.0%)	1 (4.8%)		1 (1.8%)	3 (4.1%)	
Permanent pacemaker implantation	1 (7.7%)	1 (4.8%)		1 (1.8%)	0 (0.0%)	
Reoperation for mitral valve	0 (0.0%)	1 (4.8%)		0 (1.8%)	0 (0.0%)	
Death during follow-up	0 (0.0%)	3 (14%)	.27	9 (16%)	14 (19%)	.817
Readmission for heart failure	3 (23%)	5 (24%)	1.000	13 (23%)	17 (23%)	1.000

*TR*, Tricuspid regurgitation; *LAA*, left atrial appendage; *CABG*, coronary artery bypass grafting; *PFO*, patent foramen ovale; *ASD*, atrial septal defect.

Concomitant procedures were frequently performed in both groups (Table 2). Mitral valve surgery was the most common concomitant procedure, performed in 74% of patients with severe TR and 73% of those with massive/torrential TR. Isolated tricuspid valve surgery without concomitant valve intervention was performed in 34 patients. There were no significant intergroup differences regarding prevalence of concomitant procedures or operation-related times.

The aortic crossclamp time reflects the high prevalence of concomitant procedures, particularly mitral valve surgery. Among the 22 patients undergoing isolated tricuspid valve surgery, only 4 underwent annuloplasty alone, whereas the majority required valve replacement or additional complex repair techniques.

### Postoperative Outcomes

Postoperative outcomes show in Table 2. Two patients with massive/torrential TR (2.1%) died (arrhythmia, low output syndrome) within 30 days of surgery, whereas no deaths occurred among patients with severe TR (*P* = .510). Within 30 days after surgery, stroke occurred in 2 patients, perioperative myocardial infarction in 3 patients, and low output syndrome in 2 patients. Re-exploration for bleeding was required in 5 patients. Permanent pacemaker implantation was performed in 3 patients. One patient underwent mitral valve replacement for residual mitral regurgitation.

During the follow-up examinations period, a total of 26 died (9 in severe, 17 in massive/torrential group), whereas 38 patients were readmitted for heart failure (16 in severe, 22 in massive/torrential group). The main cause of death during follow-up was heart failure (n = 6, 23%), followed by infection (n = 4, 15%), malignancy (n = 3, 12%), and renal failure (n = 2, 8%).

**Figure 2:**
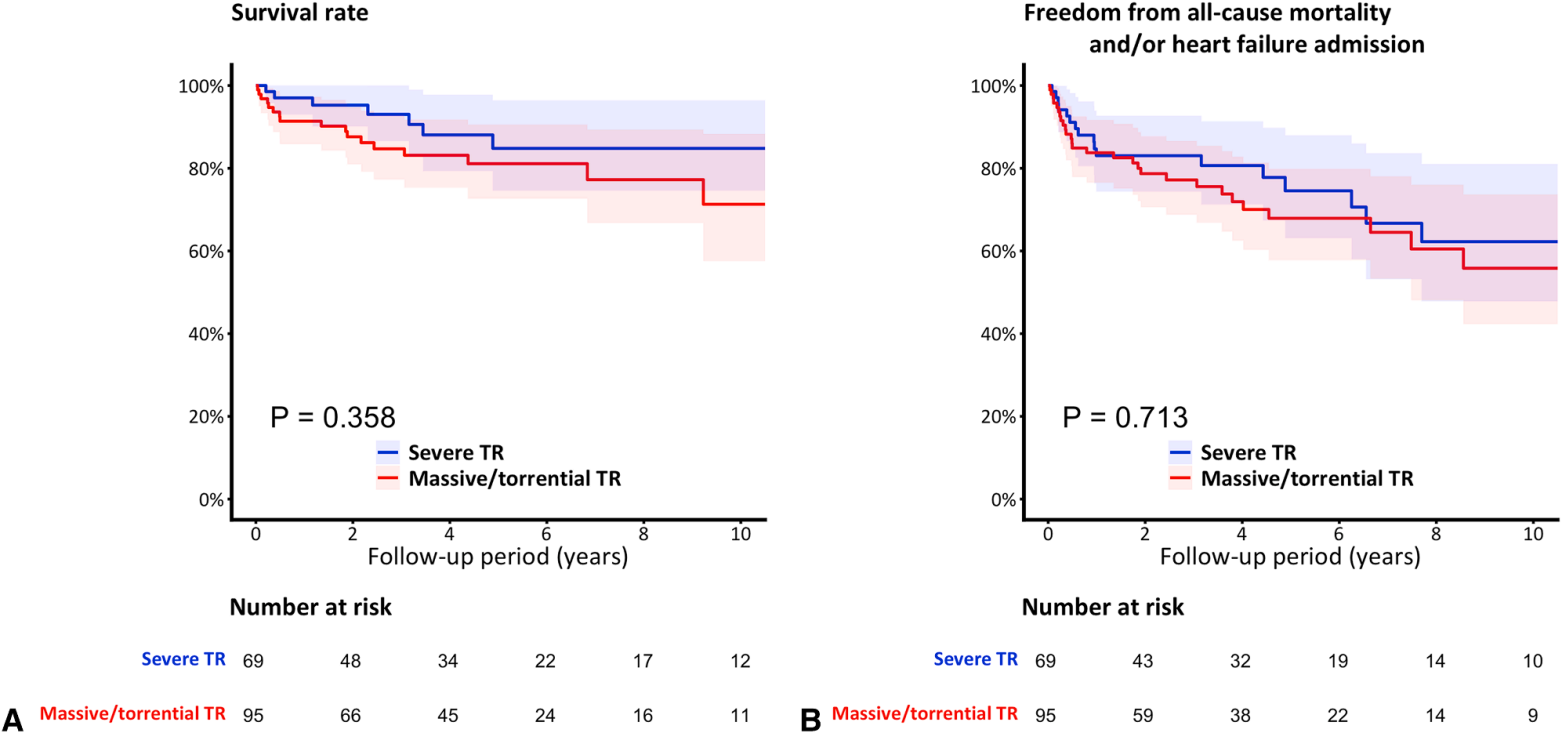
Kaplan-Meier curves with 95% CIs for (A) survival rate and (B) rate of freedom from all-cause mortality and/or heart failure, with patients divided according to TR severity (severe or massive/torrential). *TR*, Tricuspid regurgitation.

The 5-year survival rate was 85% in the severe group and 81% in the massive/torrential group, which was not a significant difference (*P* = .358) (Figure 2, *A*). Similarly, freedom from the composite end point of death or heart failure readmission did not differ significantly between the groups (75% vs 68%, *P* = .713) (Figure 2, *B*).

### Serial Echocardiographic Assessments

**Figure 3:**
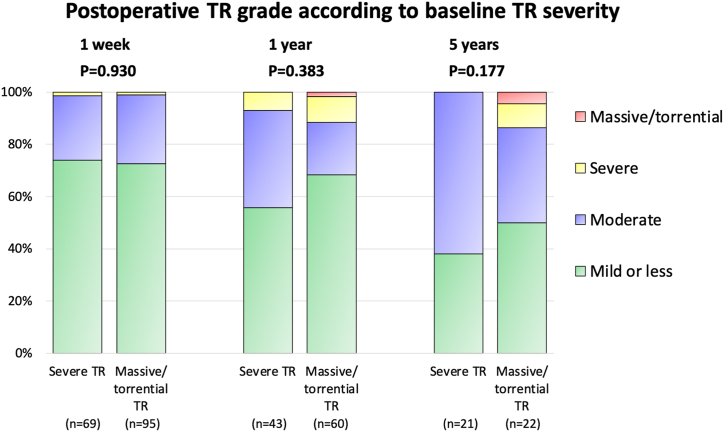
Postoperative TR grading at 1 week, 1 year, and 5 years after surgery according to the severity of TR (severe or massive/torrential). *TR*, Tricuspid regurgitation.

At the 1-week postoperative assessment, TR severity was significantly improved in both groups, with comparable rates for residual TR mild or less (73.9% vs 72.3%, *P* = .930). Thereafter, the proportion of patients with residual TR mild or less gradually decreased over time, though the intergroup differences remained nonsignificant after one year (54.5% vs 70%, *P* = .383) and also 5 years (38.1% vs 50%, *P* = .177) (Figure 3). Early recurrence or residual TR within 1 week after tricuspid valve repair was observed in 2 patients; 1 underwent early conversion to tricuspid valve replacement, whereas the other patient required late re-repair due to progression to massive TR. In addition, 4 patients developed recurrent severe TR during follow-up.

**Figure E2:**
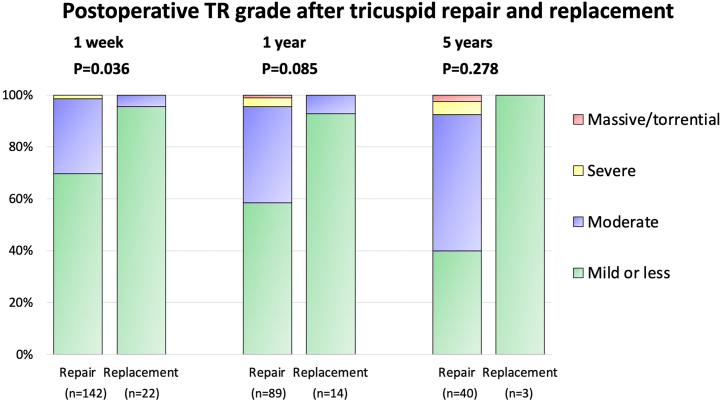
TR grade after tricuspid valve repair and replacement. *TR*, Tricuspid regurgitation.

Results of analyses comparing valve repair with valve replacement are presented in Figure E2. As for patients who underwent replacement, postoperative TR was mild or less in all except one, who developed moderate TR due to perivalvular leakage.

**Figure 4:**
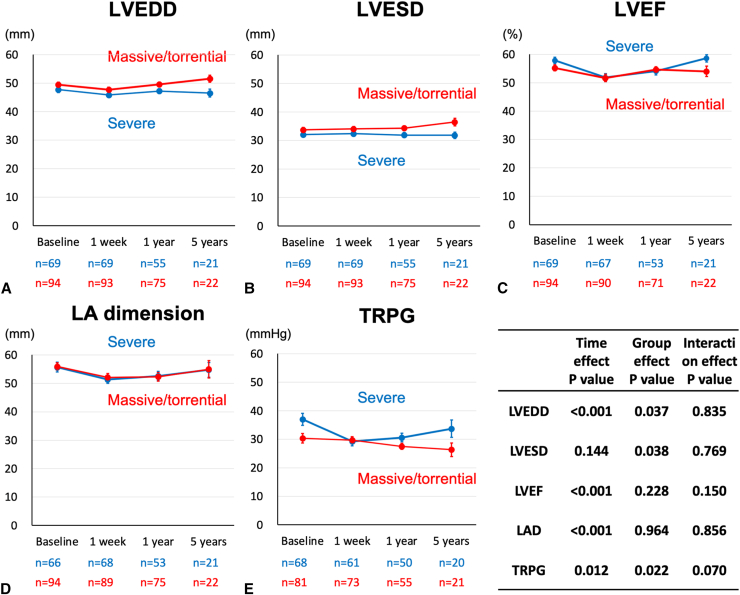
Serial changes in A, LVEDD, B, LVESD, C, LVEF, D, LA dimension, and E, TRPG. *LVEF*, Left ventricular ejection fraction; *TR*, tricuspid regurgitation; *LVEDD*, left ventricular end-diastolic dimension; *LVESD*, left ventricular end-systolic dimension; *LA*, left atrial; *LAD*, left atrial dimension; *TRPG*, tricuspid regurgitation pressure gradient.

**Figure E3:**
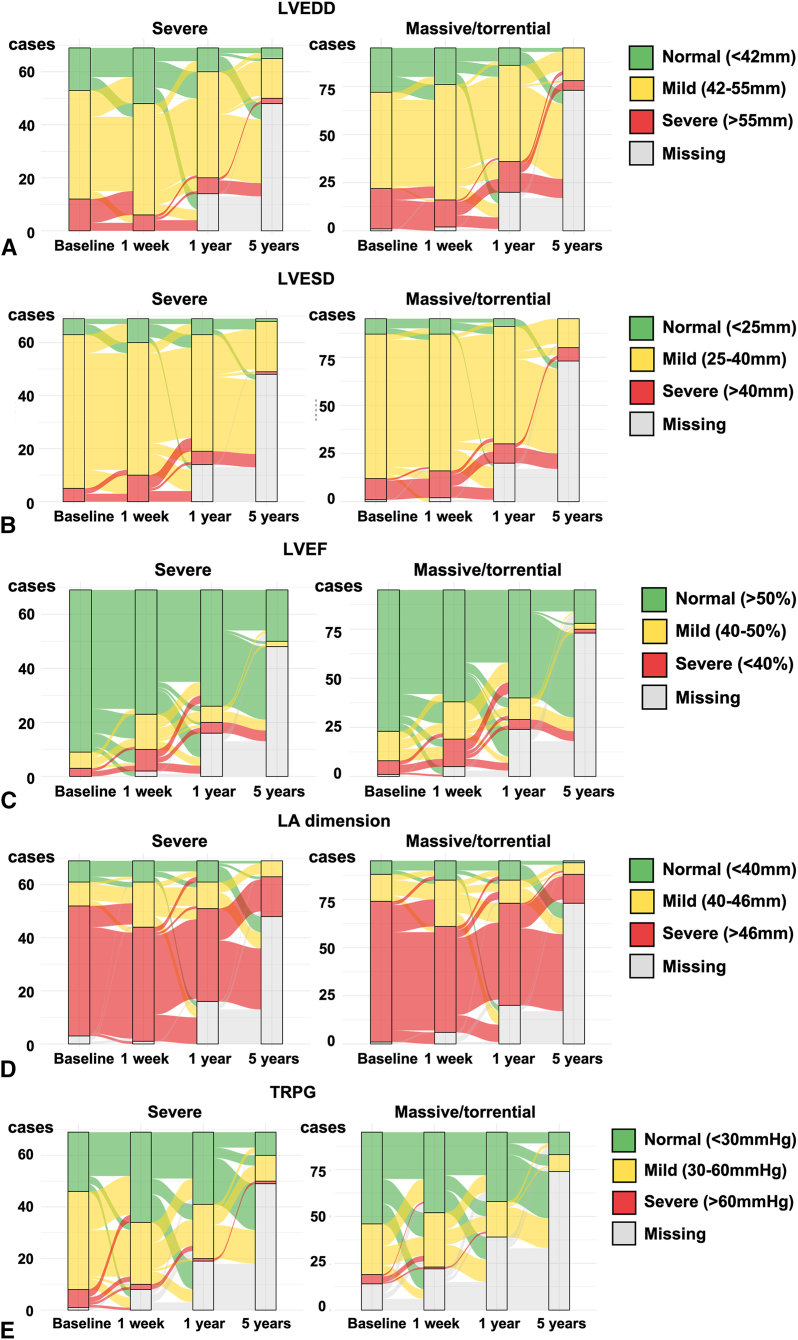
The alluvial diagram depicts patient-level transitions in tricuspid regurgitation severity at each follow-up time point. A, LVEDD. B, LVESD. C, LVEF. D, LA dimension. E, TRPG. “Missing” indicates unavailable data at the corresponding time point. *LVEDD*, Left ventricular end-diastolic dimension; *LVESD*, left ventricular end-systolic dimension; *LVEF*, left ventricular ejection fraction; *LA*, left atrial; *TRPG*, tricuspid regurgitation pressure gradient.

Serial changes in LV and left atrial dimensions, LV ejection fraction, and tricuspid regurgitation pressure gradient (TRPG) are shown in alluvial diagrams (Figure 4 and Figure E3). Early after surgery, a decrease in LV end-diastolic dimension was noted (time effect *P* < .001), whereas LV end-systolic dimension remained the same (time effect *P* = .144). LV ejection fraction showed a reduction in the early postoperative phase and then progressive recovery thereafter up to 5 years after surgery, with no differences between the groups (time effect *P* < .001, interaction effect *P* = .150). Both groups also showed a significant decrease in LA dimension in the early postoperative phase, followed by a gradual increase thereafter (*P* < .001). TRPG was gradually reduced in patients with massive/torrential TR for up to 5 years after surgery, whereas it showed a gradual increase from 1 year after surgery in those with severe TR (time effect *P* = .012, group effect *P* = .022). However, TRPG remained consistently lower compared with the preoperative baseline value in the severe TR group throughout the observation period.

**Figure E4:**
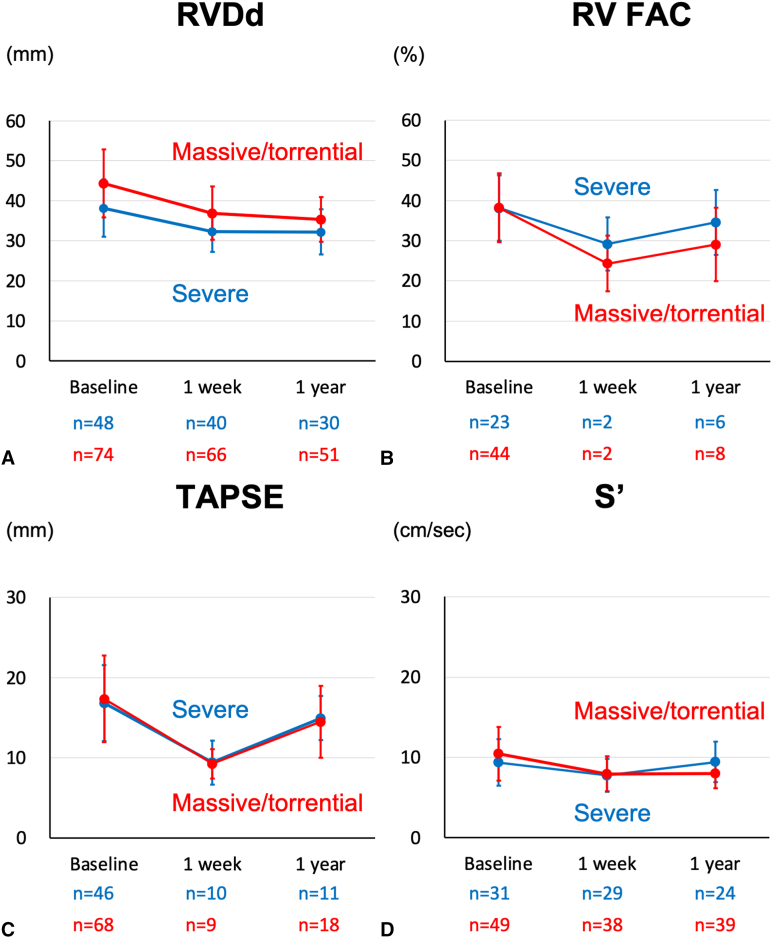
Serial changes in RV size and functional parameters. A, RVDd. B, RV FAC. C, TAPSE. D, tricuspid annular systolic velocity (S'). *RV*, Right ventricular; *RVDd*, right ventricular end-diastolic dimension; *FAC*, fractional area change; *TAPSE*, tricuspid annular plane systolic excursion.

In the supplementary analysis, RV end-diastolic dimension decreased after surgery and at 1-year follow-up (Figure E4). RV fractional area change transiently decreased in the early postoperative period but improved at 1 year, with no significant difference compared with preoperative values.

### Determinants of Adverse Events

Variables included in the multivariable models were selected on the basis of clinical relevance and statistical significance in univariable analyses. Given the limited number of events, the number of covariates in each model was restricted to minimize overfitting, and only variables shown in Table 3 were included in the final multivariable analyses.

**Table 3:** Preoperative and operative factors associated with mortality and heart failure

Variable	Univariate		Multivariate	
	Hazard ratio (95% CI)	*P* value	Hazard ratio (95% CI)	*P* value
Mortality				
Massive/torrential TR	1.46 (0.65-3.28)	.361	1.39 (0.59-3.23)	.450
LV ejection fraction, %	0.93 (0.90-0.96)	<.01	0.95 (0.92-0.98)	<.01
eGFR, mL/min/1.73 m^2^	0.95 (0.93-0.98)	<.01	0.96 (0.94-0.98)	<.01
Serum creatinine, mg/dL	1.54 (1.29-1.83)	<.01		
Hemoglobin level, g/dL	0.74 (0.61-0.90)	<.01		
Age, y	1.01 (0.97-1.05)	.570		
TVR	2.51 (0.99-6.35)	.052	1.94 (0.73-5.15)	.186
Previous cardiac surgery	1.14 (0.52-2.53)	.739		
Mortality and heart failure				
Massive/torrential TR	1.11 (0.64-1.94)	.713	1.10 (0.60-2.02)	.764
LV ejection fraction, %	0.96 (0.94-0.99)	<.01	0.97 (0.94-1.00)	.027
eGFR, mL/min/1.73 m^2^	0.98 (0.97-1.00)	<.01	0.98 (0.97-1.00)	.016
Serum creatinine, mg/dL	1.32 (1.13-1.55)	<.01		
Hemoglobin level, g/dL	0.75 (0.65-0.87)	<.01		
Age, y	1.01 (0.99-1.04)	.333		
TVR	2.37 (1.17-4.80)	.016	1.99 (0.94-4.23)	.074
Previous cardiac surgery	1.78 (1.03-3.08)	.041	1.66 (0.94-2.94)	.082

Creatinine and hemoglobin levels were significantly correlated with eGFR and thus were not included in multivariable analysis to avoid multicollinearity. *TR*, Tricuspid regurgitation; *LV*, left ventricle; *eGFR*, estimated glomerular filtration rate; *TVR*, tricuspid valve replacement.

Multivariable Cox proportional hazards analysis showed preoperative eGFR (adjusted HR, 0.96; 95% CI 0.94-0.98, *P* < .01) and LV ejection fraction (adjusted HR, 0.95; 95% CI, 0.92-0.98, *P* < .01) as independent predictors of mortality. Analysis of the composite outcome of mortality or heart failure readmission indicated that preoperative eGFR (adjusted HR, 0.98; 95% CI, 0.97-1.00, *P* = .016) and LV ejection fraction (adjusted HR, 0.97; 95% CI, 0.94-1.00, *P* = .027) remained significant predictors. Tricuspid valve replacement and history of cardiac surgery were not independently associated with long-term outcomes.

### Subgroup and Propensity-Adjusted Analyses

**Figure E5:**
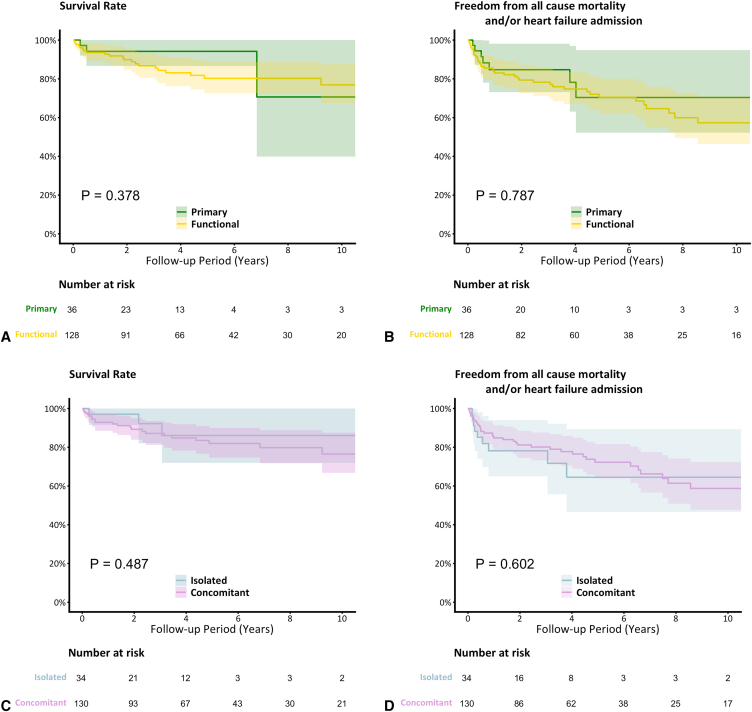
Kaplan-Meier curves with 95% CIs for survival rate and rate of freedom from all-cause mortality and/or heart failure, with patients divided according to TR etiology (primary or functional) (A and B) and isolated versus concomitant surgery (C and D). *TR*, Tricuspid regurgitation.

Given the heterogeneity of the cohort, including isolated and concomitant procedures as well as primary and functional TR, additional stratified analyses were performed. There were no significant differences in survival or freedom from the composite end point of death or heart failure readmission between primary and functional TR (Figure E5, *A* and *B*) or between isolated and concomitant procedures (Figure E5, *C* and *D*).

Kaplan-Meier analyses and stratified Cox proportional hazards models demonstrated that TR etiology (primary vs functional) was not associated with clinical outcomes (composite end point: *P* = .787; mortality: *P* = .378). Similarly, isolated versus concomitant surgery showed no significant association with outcomes (composite: *P* = .602; mortality: *P* = .487).

## Discussion

Several key findings of 164 symptomatic patients who underwent tricuspid valve surgery for significant TR, including 95 with massive or torrential TR, were noted in the present study. First, patients who underwent tricuspid valve surgery, most commonly performed for repair, showed low operative mortality (1.2%), significant postoperative reduction in TR severity, and favorable 5-year survival (85% for severe, 81% for massive/torrential TR). Furthermore, greater preoperative LV ejection fraction and better renal function were independently protective against adverse outcomes, whereas baseline TR grade itself was not associated with prognosis. These findings were further supported by stratified analyses accounting for cohort heterogeneity. Both Kaplan-Meier analyses and stratified Cox proportional hazards models demonstrated that TR etiology and the presence of concomitant procedures were not independently associated with clinical outcomes. Taken together, they indicate that surgical correction, even when performed for massive or torrential TR, is safe, effective, and yields long-term outcomes comparable with those of patients with less-advanced disease, provided that ventricular and renal functions are preserved.

The high prevalence of concomitant mitral valve surgery should be considered when interpreting these findings. Because significant TR in contemporary practice is most often secondary and associated with left-sided valve disease, the present cohort reflects real-world surgical practice rather than a highly selected isolated TR population. Previous studies have reported that TR associated with left-sided valve disease is classified as severe in approximately 25.5%, massive in 3.9%, and torrential in 1.0% of cases and that in atrial functional mitral regurgitation, severe or greater TR is present in approximately 15% of patients with moderate or greater MR and in up to one-third of those with severe MR.^14^^,^^15^ Notably, favorable survival and durable TR reduction were also observed in a separate analysis limited to isolated tricuspid valve surgery.

However, comparison between tricuspid valve repair and replacement is limited by the predominantly concomitant nature of the cohort and the lack of comprehensive preoperative right-heart hemodynamic data. Accordingly, the subgroup analysis comparing surgical strategies should be interpreted as exploratory.

Importantly, the present study is distinguished by its specific focus on patients with severe or more advanced TR, including massive and torrential grades, who have been underrepresented in previous surgical series that largely included patients with moderate or greater TR.^4^^,^^5^ By explicitly addressing this high-risk subgroup, our findings provide contemporary surgical evidence that advanced TR severity alone does not preclude favorable outcomes when patients are appropriately selected.

### Comparison With Previous Studies

Axtell and colleagues^5^ analyzed 3276 patients with isolated severe TR, of whom 171 (5%) were treated surgically (143 [84%] with repairs, 28 [16%] with replacements) and 3105 (95%) received medical treatment alone. Although those treated with surgery were younger that the patients who received medical treatment (61.0 ± 16.2 vs 73.0 ± 15.5 years), there was no significant difference in 5-year survival rates (52% in surgical group, 64% in medically treated group), indicating that surgery did not improve survival. In addition, tricuspid surgery was associated with an approximately 10% mortality rate within 30 days after surgery. Overall, poor long-term outcomes were noted in both patients who did and did not undergo surgery, with surgery providing no clear advantage related to survival. That study concluded that tricuspid valve surgery is often performed too late, after irreversible heart or organ damage, and emphasized the importance of earlier diagnosis and less invasive treatment options for severe TR. The present cohort, with 5-year survival exceeding 80% regardless of TR grade, highlights improved surgical outcomes in the present era, likely as the result of advancements in perioperative management, earlier referral, and improved repair techniques.

Our finding showing no significant survival difference between severe and massive/torrential TR is in contrast with previous registry data suggesting worse outcomes with increasing TR severity.^11^ In the TriValve Registry, patients with massive or torrential TR who underwent a transcatheter tricuspid valve intervention procedure showed a lower rate of procedural success and increased mortality compared with those with less-advanced TR.^2^ The present findings showing postsurgical improvements in early mortality rate, long-term survival, changes in LV function parameters, and TR severity suggest that surgery can mitigate the excess risk of advanced TR severity when performed for suitable cases. Furthermore, they indicate that massive or torrential TR should not considered prohibitive for operative management.

Consistent with other reports, tricuspid valve repair was found to be associated with better long-term outcomes than replacement.^16^ In the present study, tricuspid valve replacement was associated with increased risk of mortality and/or heart failure readmission in univariate analysis, with a trend toward significance after multivariable adjustment, likely reflecting greater baseline risk and anatomic complexity in patients selected for replacement. They thus reinforce current surgical recommendations to favor repair whenever feasible and restrict replacement to patients with severely degenerated or nonrepairable valves.

These results showing impaired LV ejection fraction and renal dysfunction as independent predictors of adverse events underscore the interplay among systemic congestion, ventricular-renal interaction, and overall surgical prognosis.^17^^,^^18^ In previous studies, TR has been shown to be not only a marker but also a mediator of multiorgan dysfunction, which leads to venous congestion, hepatic injury, and renal impairment. As TR progresses, chronic right-sided volume overload contributes to interventricular dependence, LV underfilling, and secondary LV dysfunction; thus, early surgical intervention before the onset of significant ventricular or renal impairment may be crucial for improving outcomes.

In the present study, most patients underwent concomitant left-sided surgery and had predominantly functional TR. In this context, left-heart structure and function represent important contributors to TR pathophysiology. Given the limited availability of invasive hemodynamic data, longitudinal changes in left-heart echocardiographic parameters were used as surrogate markers of postoperative cardiac function. Notably, these parameters demonstrated similar postoperative trajectories across TR severity groups, suggesting comparable left-heart functional responses after surgery despite differences in baseline TR grade. Despite similar postoperative trends, left atrial size and TRPG remained elevated over time, possibly reflecting irreversible remodeling attributable to chronic pulmonary hypertension or RV dysfunction, which may contribute to heart failure events after TR correction.

Interestingly, baseline TR grade was not associated with mortality or readmission in the present analysis. This suggests that surgical correction of regurgitation can reverse the adverse hemodynamic consequences of massive or torrential TR, provided end-organ damage is limited. These results align with recent findings showing that timely surgical or transcatheter correction can restore forward flow and arrest progressive RV remodeling.^19^

### Clinical Implications

This study provides several important insights for the contemporary management of advanced TR. First, the results support expansion of surgical indications to include patients with massive or torrential TR, because surgical repair can be safely performed in such cases with excellent midterm outcomes. Second, early referral before the development of irreversible LV or renal dysfunction, parameters with prognostic importance, is essential.^13^ Third, repair should remain the default surgical strategy, because optimal annuloplasty, leaflet augmentation, and chordal techniques enable sustained TR reduction, as shown by the high rates of early residual mild or less TR in both groups. Finally, these findings are considered to provide an important benchmark for the rapidly evolving field of transcatheter tricuspid valve intervention. Although such transcatheter approaches have demonstrated favorable procedural safety, registry data indicate suboptimal efficacy and greater residual TR in massive/torrential cases.^11^ The present results indicating 5-year survival >80% and durable TR reduction underscore surgery as the gold standard for operable patients, particularly those with an acceptable risk profile and reparable anatomy.

### Limitations

This study has several limitations. It was performed as a retrospective analysis of single-center findings with inherent selection bias, and repair or replacement decision-making was at the discretion of the individual surgeons. Although the mean follow-up duration was substantial (4.6 ± 3.9 years), further study of longer-term outcomes, including late TR recurrence and RV function, is warranted. Assessment of TR severity was determined solely by vena contracta width, because comprehensive multiparametric echocardiographic data were not consistently available in all patients. Although this classification method has been validated in previous studies of massive and torrential TR, the absence of additional quantitative and 3-dimensional parameters represents a limitation of the present study. Data for quantitative RV parameters, such as tricuspid annular plane systolic excursion or RV strain, were not available for all patients. Because the assessment of RV function is an important factor in clinical decision-making, the absence of comprehensive RV functional data may have influenced surgical indication and patient selection, potentially introducing selection bias. Importantly, the absence of comprehensive RV functional metrics limits the ability to disentangle the independent prognostic impact of TR severity from underlying RV dysfunction. Therefore, TR grade in this study may partially reflect advanced RV disease rather than serving as an entirely independent driver of outcomes.

In addition, invasive hemodynamic data from right heart catheterization, including cardiac output, stroke volume, pulmonary artery pressure, and pulmonary vascular resistance, as well as detailed perioperative medical therapy data, were not consistently available. Furthermore, the severity of TR is known to be influenced by preload and afterload conditions at the time of echocardiographic assessment. The lack of systematically available invasive hemodynamic data limits the ability to interpret echocardiographic findings within their physiological context. Although survival status or last clinical contact was available for all patients, the number of patients at risk decreased at later follow-up intervals because of limited follow-up duration in more recent cases and the occurrence of clinical events. Echocardiographic data at 5 years were available only in a subset of patients, because routine imaging was not consistently performed in clinically stable and asymptomatic individuals. Finally, comparisons with medical or transcatheter treatment cohorts were not performed; thus, these findings should be interpreted within the context of patients who were treated surgically.

## Conclusions

Surgical correction of TR, severe as well as massive or torrential, can be performed with low perioperative mortality and favorable long-term outcomes. Prognosis is primarily determined by baseline LV ejection fraction and renal function rather than preoperative TR grade. The present findings highlight the importance of early surgical referral and repair before the onset of irreversible organ dysfunction. In addition, they provide solid evidence supporting surgery as effective treatment for even the most advanced forms of TR.

## Conflict of Interest Statement

The authors reported no conflicts of interest.

The *Journal* policy requires editors and reviewers to disclose conflicts of interest and to decline handling or reviewing manuscripts for which they may have a conflict of interest. The editors and reviewers of this article have no conflicts of interest.
